# Influence of Tumor Site on Survival in Young Patients with Head and Neck Squamous Cell Carcinoma

**DOI:** 10.3390/curroncol29020082

**Published:** 2022-02-10

**Authors:** Claudius Steffen, Iris Piwonski, Max Heiland, Carmen Stromberger, Grzegorz Kofla, Christian Doll, Annekatrin Coordes, Benedicta Beck-Broichsitter

**Affiliations:** 1 Department of Oral and Maxillofacial Surgery, Charité—Universitätsmedizin Berlin, corporate member of Freie Universität Berlin and Humboldt-Universität zu Berlin, 13353 Berlin, Germany; max.heiland@charite.de (M.H.); christian.doll@charite.de (C.D.); benedicta.beck-broichsitter@charite.de (B.B.-B.); 2 Department of Pathology, Campus Mitte, Charité—Universitätsmedizin Berlin, corporate member of Freie Universität Berlin and Humboldt-Universität zu Berlin, 10117 Berlin, Germany; iris.piwonski@charite.de; 3 Department of Radiooncology, Campus Virchow-Klinikum, Charité—Universitätsmedizin Berlin, corporate member of Freie Universität Berlin and Humboldt-Universität zu Berlin, 13353 Berlin, Germany; carmen.stromberger@charite.de; 4 Department of Oncology, Campus Virchow-Klinikum, Charité—Universitätsmedizin Berlin, corporate member of Freie Universität Berlin and Humboldt-Universität zu Berlin, 13353 Berlin, Germany; grzegorz.kofla@charite.de; 5 Department of Otorhinolaryngology, Head and Neck Surgery, Campus Virchow-Klinikum and Campus Charité Mitte, Charité—Universitätsmedizin Berlin, corporate member of Freie Universität Berlin and Humboldt-Universität zu Berlin, 13353 Berlin, Germany; annekatrin.coordes@charite.de

**Keywords:** squamous cell carcinoma of head and neck, head and neck neoplasms, survival, young adult, child, cancer, risk factors

## Abstract

The number of patients under the age of 45 diagnosed with head and neck squamous cell carcinomas (HNSCC) is increasing, probably due to the incidence of oropharyngeal cancers. Comparisons of HNSCC in young and old patients regarding tumor site and survival in sample sizes of relevance are rarely published. The aim of the study was to analyze the differences in survival between age groups dependent on tumor site and the influence of oropharyngeal cancers on the rising rates of HNSCC in the young. The records of 4466 patients diagnosed with HNSCC were reviewed retrospectively. Patients younger than 45 years were divided further into four subgroups for specific age differences in the young. The influences of patient and clinicopathological characteristics on survival were assessed using Kaplan–Meier analyses. Among the patient cohort, 4.8% were younger than 45 years. Overall survival (OS) in these patients was better, with a 5-year OS of 66.1% (vs. 46.4%), while relapse-free survival (RFS) was better in the older patient population, with a 5-year RFS of 74.9% (vs. 68.1%). Decreased RFS in the young was found for advanced tumor stages and tumor sited at the larynx. Hypopharynx and advanced stages were independent risk factors for OS under 45 years. Overall, 44.4% of all HNSCC in patients under 30 years were nasopharyngeal cancers, and incidence decreased with age. The incidence of oropharyngeal cancers increased significantly with age. Better OS in the young may be explained by lower tumor and disease stages, whereas oropharyngeal tumors and HPV were not found to cause rising rates of HNSCC. Laryngeal malignancies in young patients might be related to an increased malignant potential and should, consequently, be treated as such.

## 1. Introduction

The association of head and neck squamous cell carcinomas (HNSCCs) with extrinsic risk factors such as smoking and alcohol is well known [1]. About 50–70% of all HNSCC patients consume alcohol or tobacco [1], resulting in a mean age at diagnosis of around 64 years [2]. In the age group of patients younger than 40 years, long-term exposure to these risk factors is very rare, and consequently, the proportion of HNSCCs in this group compared to the overall incidence of HNSCC is at a low level of around 2.7% [3] to 6% [4]. Although the development of incidences of HNSCCs varies between different countries, there is a trend toward higher incidences of HNSCC in patients younger than 45 years [5].

Several studies indicate a dependency on the site of tumors in these cases. One study could demonstrate increasing rates exclusively in SCCs of the tongue and tonsils [6]. Another study also showed a higher proportionate increase in tongue cancer in young patients than in older ones [7]. On the other hand, incidences of SCCs localized at the larynx (10%) [8] or hypopharynx (1%) [9] showed constant rates in the young age group [1]. Given that the overall incidence of SCC in all age groups has been decreasing slightly over the past decades while increasing rates of tumors of the oropharynx were found [10], one might assume a causal relationship between the increasing rates of oropharynx tumors and the increasing numbers of HNSCCs in the young patient cohort.

Different distributions of risk factors in age groups might be an explanation for differences in tumor sites between age groups. Several reviews suggest factors other than smoking and alcohol to be of relevance in the young [1,4,11]. These might be of genetic origin, e.g., Li–Fraumeni syndrome or Fanconi’s anemia [12]. Furthermore, the human papillomavirus (HPV) was identified as increasingly prominent [11], which might explain the increasing numbers of oropharyngeal cancer in patients below the average age of around 64 years [10]. Whether this also affects patients under the age of 45 years to a relevant degree is unclear, however.

Several previous studies analyzed patients aged under 45 years in order to reveal the impact of clinicopathological factors on survival compared to older patients. Some studies could demonstrate better disease-free survival rates in young patients [13,14], whereas others could not show any significant differences [15,16]. The problem of most studies involving young patients is small case numbers, which may reduce the validity of many studies [1]. In addition, to the authors’ knowledge, no recent study has ever performed a detailed age-categorized analysis of the young patient group itself in a single-center cohort with relevant case numbers.

For this reason, our objectives were to (1) analyze survival rates and tumor distributions in patients aged under 45 years compared with older patients without bias produced by multiple institutions or time periods; (2) evaluate possible differences in frequencies of tumor sites, as well as clinicopathological factors, between young patients and older patients; and (3) examine the influence of these differences on survival with increasing ages also using a more detailed aged-categorized analysis of the young patient group itself. A retrospective analysis of our single-center data of a large sample of HNSCC patients within a 10-year period was performed in order to address these objectives.

## 2. Materials and Methods

### 2.1. Patient Inclusion Criteria

This study was approved by the Ethics Committee of the medical faculty of Charité —Berlin (EA1/256/20). All patients referred to the Charité—University Medical Center Berlin with primary histopathologically confirmed HNSCC between 2009 and 2018 were included in this study. Subsequently, only patients diagnosed at the age of 44 years or younger were subjected to a more detailed analysis. Patients with tumor locations other than the head and neck or initial tumor treatment at another institution were excluded from the analysis.

### 2.2. Data Collection and Processing

Age, gender, date of diagnosis, date of death or relapse, date of last patient information, American Joint Committee on Cancer (AJCC) stage, and tumor site data were collected retrospectively. Patients aged under 45 years were further categorized into four groups: 0–29 years, 30–34 years, 35–39 years, and 40–44 years. They were included in a further analysis that incorporated all previously indicated information, as well as TNM status, treatment modality, and p16 status of all tumors located at the oropharynx.

OS was defined as the time between first histological diagnosis and date of death or last contact with patients, reviewed until December 31, 2019. RFS was the time between initial diagnosis and date of recurrence or, in the case of no recurrence, date of last contact or death.

Tumor staging and grading was performed according to the eighth edition of the AJCC [17]. In oropharynx tumors with missing p16 status, the seventh edition of the AJCC was used [18]. Each case was discussed in a multidisciplinary tumor conference, and treatment proposals were made according to international standards at the time of diagnosis. Postoperative follow up was done at 1, 2, 3, 6, 9, 12, and 15 months, and subsequently, every 6 months for 5 years post-treatment. Follow up consisted of clinical examinations and magnetic resonance imaging (MRI) or computed tomography (CT) at specific intervals.

### 2.3. p16 Status

Immunohistochemical staining of the formalin-fixed and paraffine-embedded tissue samples was performed using the BenchMark ULTRA Autostainer (Ventana, Tucson, Arizona, USA) and monoclonal rabbit p16 antibodies INKA4 (CINtec Histology Kit; Ventana Medical Systems Inc., 1910 E. Innovation Park Drive Tucson, Arizona 85755, USA) according to the manufacturer’s instructions.

A positive p16 status was defined by a medium-to-strong (2+/3+) intensity of nuclear staining with a distribution of ≥75% (of the tumor cells). Cytoplasmatic staining was of no relevance.

### 2.4. Statistical Analysis

Statistical analysis was performed using IBM SPSS Statistics Version 26.0 (IBM Corporation, Armonk, NY, USA). Qualitative variables were demonstrated with frequency and percentage, quantitative variables with mean and standard deviation. Survival was presented using the Kaplan–Meier method. The primary outcomes were the OS and RFS of young patients compared with that of patients aged older than 45 years. Secondary outcomes were differences in OS and RFS related to tumor sites between both age groups and between categorized age groups under 45 years. A *t*-test was used for direct comparison of variables for normally distributed data, and the Mann–Whitney-U-test was used for non-normally distributed data. The chi-squared test was used for direct comparison of qualitative variables. Distributions of survival were compared using log-rank tests. Multivariate survival analyses were compared using Cox regression analysis with backward elimination. The level of statistical significance was set as *p* < 0.05.

## 3. Results

### 3.1. Patient Characteristics

In all, 4466 patients diagnosed with HNSCC with a median age at diagnosis of 63.0 (range 14–106) years and a median follow-up time of 22.0 (0–100) months, were included. Of this sample, 4.8% (*n* = 215) were younger than 45 years, with a median follow up of 29.0 (0–100) months and a median age of 41.0 (14–44) years. The control group (≥45 years) included 4251 patients with a median follow up of 21.0 (0–100) months and a median age of 64.0 (45–106) years. In both age groups, most tumors were localized in the oral cavity, followed by oropharynx tumors.

Oropharynx tumors had a proportionally lower incidence in the young age group. Conversely, nasopharynx tumors occurred in higher proportions in younger than in older patients The distribution of tumor sites was significantly different between both groups (*p* < 0.001) (Table 1).

Tumors were categorized into early stage disease (AJCC I/II) and advanced-stage disease (AJCC III/IV). In both age groups, advanced stages were represented by a higher number of patients. The number of cases of advanced-stage disease was significantly lower in young patients (*p* = 0.002). Distribution of sex did not differ between both groups (*p* = 0.150). The p16 status of patients aged under 45 could be investigated in 21 of 43 patients (53.5%) with oropharyngeal tumors; 54.2% of these had a positive p16 status.

### 3.2. Survival Outcomes

**Figure 1:**
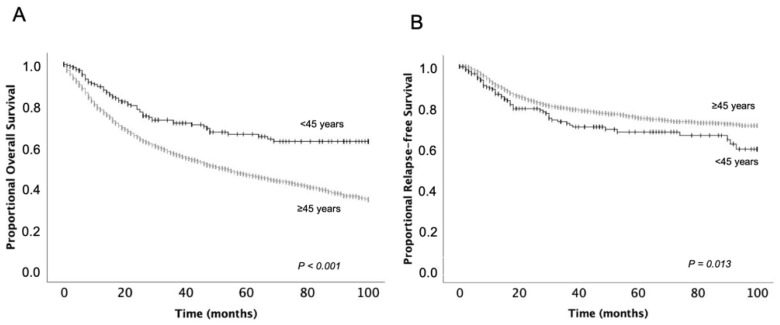
Overall survival (**A**) and relapse-free survival (**B**) of patients aged under 45 years in comparison to older patients.

The mean OS for all patients was 56.0 (±0.7) months, and the mean RFS was 79.2 (±0.7) months. Young patients had a significantly higher OS than older patients (Table 2, Figure 1). However, RFS was significantly lower in younger patients (Table 2).

**Figure 2:**
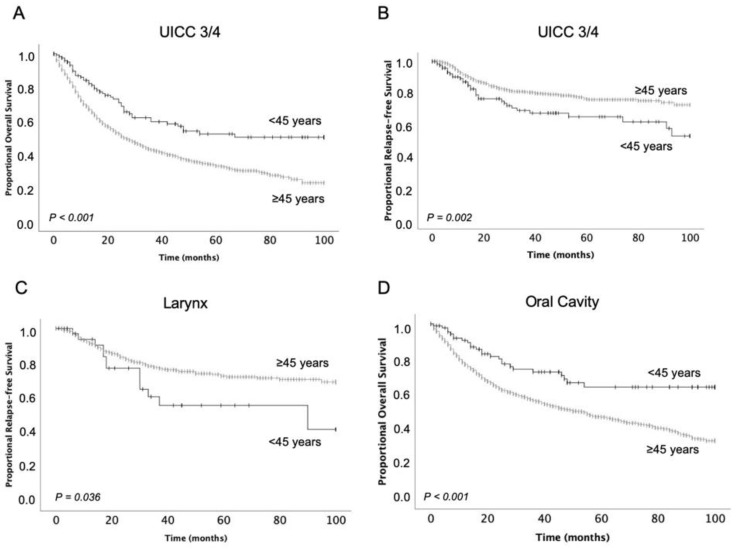
Overall survival (**A**) for advanced disease stages and tumors localized at the oral cavity (**D**) and relapse-free survival for advanced disease stages (**B**) and tumors localized at the larynx (**C**) in patients aged under 45 years in comparison to older patients.

The 1-year, 3-year, and 5-year OS of patients under 45 years were 89.2%, 71.5%, and 66.1%. In patients over 45 years, these rates were 78.0%, 56.7%, and 46.4%, respectively. The corresponding values for RFS were 86.6%, 72.3%, and 68.1%, respectively, for patients younger than 45 years, and 91.0%, 79.5%, and 74.9% for older patients. OS was significantly higher in both early and advanced stages in young patients. RFS was significantly lower in younger patients with advanced stages, but at the early stages, RFS did not differ significantly between young and old patients (Figure 2, Table 2).

OS survival was dependent on tumor site in both younger (*p* = 0.001) and older patients (*p* < 0.001). RFS was also significantly dependent on tumor site (*p* < 0.001) in older patients but not in young patients under 45 years (*p* = 0.167).

Of all tumor sites, only oral cavity, oropharynx, and nasopharynx tumors demonstrated significantly higher OS in younger patients. The poorest OS rates were found for malignancies of the hypopharynx in both groups. The best OS survival in young patients was found for nasopharynx tumors, whereas in older patients, larynx tumors were linked to the best outcome. The larynx was the only tumor site with a significance concerning overall lower RFS in younger patients (Figure 2). Lower RFS in young patients was also demonstrated in advanced stages, but advanced stages were not represented by a higher proportion of larynx tumors in young patients than in old patients (Table 3).

Excluding larynx tumors from the general analysis, the RFS was still higher in older patients, but this difference was no longer significant (85.5% vs. 77.5%, *p* = 0.081). In both age groups, the lowest RFS was found in paranasal sinus tumors.

### 3.3. Factors Affecting Survival in Patients under 45 Years

The number of patients in each subcategory of patients aged younger than 45 years increased with age. OS and RFS did not differ significantly between categorized age groups of patients aged under 45 years (Table 4).

Regardless of tumor site, 61.5% of younger patients were treated by surgery. In 38.5% of cases, a non-surgical approach was chosen. For each tumor site, there was one patient receiving palliative treatment; overall, such patients represented 2.9% of all young patients. In young patients, 72.2% of larynx tumors were treated by surgery and 27.8% with a non-surgical approach. For hypopharynx tumors, this rate was 36.4% vs. 63.6%; for nasopharynx tumors, 0% vs. 100%, for oral cavity tumors, 78.9% vs. 21.1%; and for oropharynx tumors, 55.0% vs. 45.0%.

In the univariate analysis of exclusively categorized age groups under 45, worse OS was associated with tumor site at the hypopharynx, higher T-stages, advanced disease, positive lymph nodes, and patients receiving primary radio(chemo)therapy (CRT) or therapy other than surgery only (Table 4). The multivariate analysis only revealed advanced stages and tumor site at the hypopharynx as independent risk factors of OS in young patients. The univariate analysis did not reveal any significant differences in RFS.

**Figure 3:**
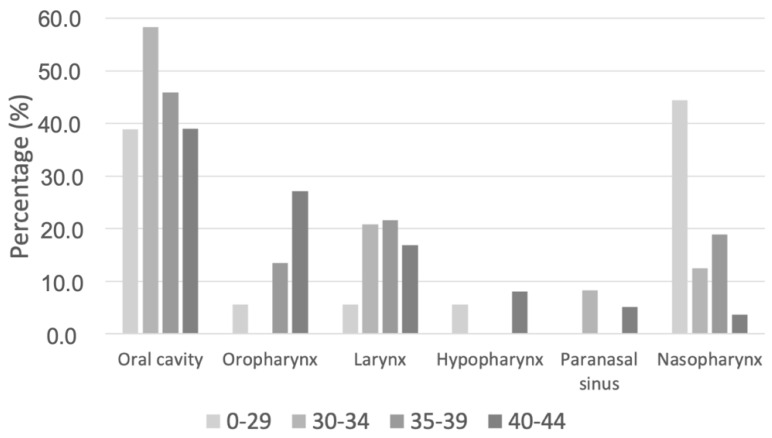
Distribution of tumor subsites in age-categorized patients under 45 years.

In patients younger than 45 years, the frequency of oropharynx tumors increased significantly with age. The occurrence of nasopharynx tumors was less likely with increasing age (Figure 3).

In the comparison of OS exclusively among classified age groups under 45 years, tumor stages 3/4 and grade 1 demonstrated significant differences. The RFS among classified age groups under 45 years did not differ in relation to any of the variables analyzed.

## 4. Discussion

In total, 4466 patients were included in this study. In accordance with the literature, 4.8% of all patients were under the age of 45 years; previous studies described a range of around 2.7% [3] to 6% [4]. SCCs are known to be influenced strongly by external factors such as smoking, alone or in combination with alcohol [19]. Due to the retrospective study design, there was no detailed information about these factors available for the young age group, and they were consecutively excluded from this study.

Although some studies suggest traditional risk factors also in the young [20], previous studies suggested other factors as risk factors for the development of HNSCCs in this age group [21]. Some inherited syndromes, which show changes in causative genes for DNA repair and surveillance of genetic stability, are known. In addition to Fanconi anemia, these are, for example, Bloom’s syndrome and Li–Fraumeni syndrome. [1] Thus far, there is little knowledge about differences in patient and tumor characteristics between young and old patients. Due to the possibly differing tumor genesis between these age groups and tumor sites, the clinical results of this study may serve as a basis for further investigations concerning the individual treatment of HNSCCs.

Differences in disease stages might have influenced better OS in young patients. Although slightly under the threshold for significance, exclusive analysis of patients under 45 years indicated that the proportion of early stage disease decreased with age and that there was a higher amount (*p* = 0.002) of early stage disease in younger patients. Young patients might be diagnosed earlier, presumably due to a higher overall disease awareness and attention concerning physical changes, which might have affected the OS in this study. There is no agreement on this question in the literature. Some studies did not compare disease stages due to matched analyses [22], while other early studies found higher disease stages in the young [3]. Most recent reviews presume similar disease stages between young and old [1,5].

In the young, advanced-stage disease was an independent risk factors impacting survival. The negative impact of advanced tumor stages for all ages is well known [23]. Notably, however, both early stage and advanced disease had significantly better OS in the young. Presumably, fewer comorbidities and complications involving the treatment support better OS [24], but it may also be caused by differences in the grade of differentiation. In the present study, only 16.7% of patients under 30 years were classified as having grade 3 tumors, and in contrast to older age groups, a higher number of tumors were classified as grade 1 (Table 4). Tsukuda et al. also found a higher number of low-grade carcinomas in young patients, although the sample size was very small [25]. In the present study, the grade of differentiation was only known for patients under 45 years, therefore comparisons to older patients could not be performed. Other studies report no difference in tumor grade between the young and the old [26,27]. The question of the influence of tumor grading on better OS in the young cannot be solved by this study.

In general, the analysis of OS may distort the findings. In older patients, OS might be influenced to a greater extent by factors such as secondary diseases. Hence, the investigation of disease-specific survival (DSS) would reduce bias. The literature is discordant concerning DSS in the young [1,4]. Studies have weaknesses either due to missing long-term survival [14], matched-pair analyses [13], or small case numbers [22]. The large case number presented by our study might reduce these weaknesses, but to answer this question, prospective multi-institutional studies are required [1].

In contrast to OS, RFS was significantly worse in young patients. This is particularly remarkable since a lower proportion of advanced disease was seen in younger patients and higher tumor stages are known as independent risk factors of tumor relapse [28]. Nevertheless, especially advanced tumor stages resulted in significantly worse RFS in young patients, whereas in early stage diseases there were no differences. Other than for advanced stages, lower RFS in young patients was only found for tumors sited at the larynx. However, there was no correlation between advanced stages and larynx tumors because further analyses revealed no overrepresentation of advanced tumor stages in the larynx in young patients. When larynx tumors were excluded from the general analysis, RFS was no longer significantly lower in younger patients. This seems to suggest that larynx tumors are an independent risk factor for relapse in young patients, but multivariate analyses could not confirm this assumption. However, worse RFS in younger ages has not been described for this tumor site before, and multicenter studies are needed to support this hypothesis. Clinically, these findings may suggest the need for a revision of treatment modalities of larynx tumors in young patients. Currently, treatment of these tumors includes surgery or radiochemotherapy [29]; 72.2% received surgery in this study. In early stage laryngeal tumors, surgery has been found to be superior compared to radiation therapy [30]. High relapse rates exclusively in larynx cancers indicate that presumably a more aggressive treatment strategy must be chosen, especially in the young. They are likely to have fewer comorbidities and may, therefore, also benefit from concomitant treatments with for example radiochemotherapy or induction chemotherapy with cisplatin, 5-fluorouracil, and docetaxel (TPF) to a greater extent [31,32].

In contrast to larynx cancers, hypopharyngeal cancers were identified as independent risk factors for OS in the multivariate analysis in patients under 45 years. Hypopharyngeal cancers are known to have the worst outcome of all HNSCCs due to late diagnosis [33], which is also underlined by the results of this study for both age groups. Some clinicians suggest that in advanced hypopharynx tumors, there might not be a difference in survival between surgery or radiochemotherapy as treatments [34]. The rate of surgical therapy for this tumor site in the young was 36.4%, compared to a surgery rate of 61.5% for all tumor sites together. Surgical therapy of these tumors is difficult due to the anatomic region and the challenge of complete tumor resection. This often results in high postoperative morbidity [35]. The present study is not able to reveal the best treatment modality for each tumor site.

The tumor site was also notable in examinations of nasopharyngeal carcinomas. In the present study, patients below 30 years accounted for 8.0% of all nasopharyngeal carcinomas diagnosed, and there was a significant trend of decreasing incidence of this tumor site with increasing ages. Given that patients under 30 years only accounted for 0.4% of all patients, nasopharyngeal carcinomas occupied a prominent position in this population. Incidences of nasopharyngeal carcinoma have been found to be dependent on geographical regions and are known to peak at the ages of 10–20 in the North American and Mediterranean populations [36]. In Southeast Asia, nasopharyngeal carcinoma is endemic, and its age peak in these populations is around 50–60 years [37]. These results, therefore, underline the relevance of this tumor site in very young patients in the German population as well. The 5-year OS of these patients under 45 years in this study was 88.4%, and nasopharyngeal carcinomas had the best OS of all tumor sites in this age group. The survival rate is in concordance with the literature showing a 5-year OS of 75–80% [38].

As initially stated, there have been reports on rising incidences of HNSCC in patients younger than 45 years [5], while increasing rates of oropharynx tumors have been described in patients aged below the average (around 60 years) [10]. Following oral tumors, oropharynx was the second most frequent tumor site in both age groups, but only 20.0% of tumors in young patients were attributed to the oropharynx, compared to 31.6% in older patients. The exclusive analysis of patients under 45 years demonstrated a significant trend in patients under 45 years toward higher numbers of oropharynx tumors at higher ages. The current study does not deliver any information about the incidences of oropharynx tumors at ages above 45 years and, therefore, does not allow a general statement concerning higher rates of oropharynx tumors at ages below the average. However, the data suggest that it is unlikely that oropharynx tumors in patients aged below 45 years are responsible for the overall increase of the HNSCC incidence this group. This is underlined by the p16 status of young patients. Only 54.2% of oropharyngeal carcinomas in young patients showed p16 overexpression. With an HPV proof of more than 70% in oropharyngeal cancers [10,11,39] in all age groups, HPV does not seem to influence the development of oropharyngeal carcinoma in the young age group to a significant degree. A limitation of this study is that less than 50% of patients with oropharyngeal cancers could undergo p16 testing. However, the latency period between HPV infection and cancer development usually lasts more than 10 years, which further suggests a limited influence in young patients [40].

## 5. Conclusions

OS was better and RFS worse in patients under 45 years. Better OS may be explained by lower tumor and disease stages. Advanced tumor stages and tumors localized at the larynx, in particular, caused lower RFS in the young; therefore, future studies may focus on new treatment modalities that address these factors. The previously demonstrated high incidence of nasopharyngeal cancers in some populations in patients aged below 30 years can now also be proven for the Central European population. Oropharyngeal tumors in young patients have a lower proportional share compared to those in old patients. This reduces the probability of a general influence of oropharyngeal cancer and, therefore, also HPV, on the increasing overall rates of HNSCC in patients younger than 45 years.

## Figures and Tables

**Table 1:** Characteristics of patients with primary diagnosed head and neck malignancies.

Variable	Age < 45 *n* (%)	Age ≥ 45 *n* (%)	*p*
**Median age (range)**	41.0 (14–44)	64.0 (45–106)	
**All patients**	215 (4.8)	4251 (95.2)	
Females	68 (31.6)	1154 (27.1)	
Males	147 (68.4)	3097 (72.9)	
Distribution of sex			0.150
**Tumor site**			
Oropharynx	43 (20.0)	1344 (31.6)	
Oral cavity	91 (42.3)	1568 (36.9)	
Larynx	37 (17.2)	766 (18.0)	
Hypopharynx	12 (5.6)	354 (8.3)	
Paranasal sinus	9 (4.2)	142 (3.3)	
Nasopharynx	23 (10.7)	77 (1.8)	
Distribution of tumor site			<0.001
**Disease stage**			
AJCC I/II	73 (36.7)	754 (26.5)	
AJCC III/IV	126 (63.3)	2090 (73.5)	
Distribution of disease stages			0.002

**Table 2:** Comparison of clinicopathologic variables associated with overall survival (OS) and relapse-free survival (RFS) in patients younger and older than 45 years.

Variable	Overall Survival			Relapse-Free Survival		
	Mean OS age <45 (months, SD)	Mean OS age ≥45 (months, SD)	*p*	Mean RFS age <45 (months, SD)	Mean RFS age ≥45 (months, SD)	*p*
**All patients**	72.1 (3.0)	55.1 (0.7)	<0.001	73.3 (3.1)	79.5 (0.7)	0.013
Females	72.4 (5.6)	57.8 (1.3)	0.016	67.2 (6.0)	79.6 (1.4)	0.015
Males	71.9 (3.5)	54.1 (0.8)	<0.001	75.7 (3.6)	79.5 (0.8)	0.172
**Tumor site**						
Oropharynx	75.4 (6.3)	55.2 (1.3)	0.006	84.9 (5.5)	83.7 (1.2)	0.900
Oral cavity	72.8 (4.1)	54.7 (1.1)	<0.001	70.3 (4.8)	76.8 (1.2)	0.122
Larynx	72.3 (6.8)	64.3 (1.6)	0.308	64.3 (7.4)	78.1 (1.7)	0.036
Hypopharynx	29.8 (9.2)	37.6 (2.1)	0.624	54.2 (15.5)	82.1 (2.6)	0.065
Paranasal sinus	61.0 (13.5)	56.6 (4.1)	0.525	36.0 (13.0)	72.6 (4.3)	0.487
Nasopharynx	89.7 (6.9)	53.8 (5.2)	0.008	87.3 (7.7)	88.9 (4.2)	0.979
**Disease stage**						
AJCC I/II	91.2 (3.4)	67.6 (1.8)	<0.001	75.8 (5.0)	74.8 (1.8)	0.918
AJCC III/IV	61.8 (4.1)	43.4 (1.0)	<0.001	70.2 (4.3)	81.1 (1.1)	0.002

**Table 3:** Larynx tumors in combination with categorized UICC stages in comparison to age groups.

Variable	All	Age < 45	Age ≥ 45	*p*
Larynx + UICC 1/2	502	21	481	0.459
Larynx + UICC 3/4	301	16	285	

**Table 4:** Analysis of clinicopathologic variables associated with overall survival (OS) and relapse-free survival (RFS) in the aged-categorized patient group under 45 years. SCC: squamous cell carcinoma; RCT: radio(chemo)therapy; T-classification: tumor classification; N-classification: lymph node classification; SD: standard deviation; HR: hazard ratio; CI: confidence interval.

Variable	0–29 Years *n* (%)	30–34 Years *n* (%)	35–39 Years *n* (%)	40–44 Years *n* (%)	*p* (Age Groups vs. Age Groups)	Univariate Analysis, P, HR (CI 95%) (Variable vs. Other Matching Variables Combining Age Groups)	Multivariate Analysis (Variable vs. Other Matching Variables Combining Age Groups), P, HR (CI 95%)
All patients, *n* (%)	18 (8.4%)	24 (11.2%)	37 (17.2%)	136 (63.3%)			
Mean OS (months)	100	68.4 (10.3)	70.8 (7.0)	70.1 (3.7)	0.146		
Mean RFS (months)	80.0 (9.4)	73.1 (10.4)	63.5 (7.4)	76.4 (3.7)	0.315		
Females, *n* (%)	6 (33.3%)	17 (70.8%)	11 (29.7%)	34 (25.0%)	<0.001		
Mean OS (months)	100	68.0 (11.4)	38.2 (4.2)	72.2 (7.7)	0.615	0.985	
Mean RFS (months)	54.7 (21.5)	74.1 (11.1)	26.2 (5.0)	76.1 (7.6)	0.132	0.245	
Males, *n* (%)	12 (66.7%)	7 (29.2%)	26 (70.3%)	102 (75.0%)	<0.001		
Mean OS (months)	100	38.0 (7.1)	72.4 (7.8)	69.3 (4.3)	0.291	0.985	
Mean RFS (months)	91.8 (7.8)	37.0 (9.0)	68.1 (8.4)	76.8 (4.3)	0.491	0.245	
Oropharynx, *n* (%)	1 (5.6%)	0 (0.0%)	5 (13.5%)	37 (27.2%)	0.003		
Mean OS (months)	100	N/A	69.0 (12.5)	76.1 (6.8)	0.938	0.617	
Mean RFS (months)	100	N/A	74.4 (14.0)	85.5 (5.9)	0.718	0.101	
Oral cavity, *n* (%)	7 (38.9%)	14 (58.3%)	17 (45.9%)	53 (39.0%)	0.330		
Mean OS (months)	100	49.0 (13.3)	75.3 (10.4)	73.8 (5.6)	0.075	0.760	
Mean RFS (months)	64.7 (16.1)	66.3 (13.9)	58.8 (10.6)	77.0 (5.7)	0.385	0.440	
Larynx, *n* (%)	1 (5.6%)	5 (20.8%)	8 (21.6%)	23 (16.9%)	0.484		
Mean OS (months)	100	100	37.3 (6.1)	72.0 (8.6)	0.359	0.975	
Mean RFS (months)	90 (0.0)	44.7 (6.0)	23.2 (3.8)	68.4 (9.2)	0.331	0.190	
Hypopharynx, *n* (%)	1 (5.6%)	0 (0.0%)	0 (0.0%)	11 (8.1%)	0.156		
Mean OS (months)	100	N/A	N/A	28.4 (9.1)	0.489	<0.001	0.011, 2.933 (1.285–6.692)
Mean RFS (months)	100	N/A	N/A	52.4 (15.7)	0.606	0.333	
Paranasal sinus, *n* (%)	0 (0.0%)	2 (8.3%)	0 (0.0%)	7 (5.1%)	0.290		
Mean OS (months)	N/A	100	N/A	57.6 (15.6)	0.617	0.868	
Mean RFS (months)	N/A	100	N/A	27.3 (14.2)	0.362	0.385	
Nasopharynx, *n* (%)	8 (44.4%)	3 (12.5%)	7 (18.9%)	5 (3.7%)	<0.001		
Mean OS (months)	100	100	84.5 (14.1)	43.0 (11.4)	0.473	0.112	
Mean RFS (months)	87.6 (11.1)	100	74 (0.0)	100	0.823	0.108	
Surgery only, *n* (%)	8 (47.1%)	11 (45.8%)	11 (29.7%)	42 (32.3%)	0.363		
Mean OS (months)	100	89.1 (10.1)	92.5 (7.1)	86.5 (5.0)	0.972	<0.001	0.489
Mean RFS (months)	67.8 (14.1)	75.1 (14.7)	67.7 (13.0)	76.3 (6.2)	0.672	0.817	
R(C)T + surgery, *n* (%)	1 (5.9%)	9 (37.5%)	8 (21.6%)	38 (29.2%)	0.108		
Mean OS (months)	100	27.1 (6.7)	71.1 (9.0)	76.8 (6.2)	N/A	0.985	
Mean RFS (months)	100	31.1 (7.6)	61.3 (13.1)	79.6 (6.3)	0.491	0.613	
RCT, *n* (%)	8 (47.1%)	3 (12.5%)	16 (43.2%)	45 (34.6%)	0.056		
Mean OS (months)	100	36.3 (9.5)	67.7 (11.3)	52.0 (7.3)	0.090	0.005	0.577
Mean RFS (months)	87.6 (11.1)	100	57.1 (8.8)	74.5 (7.6)	0.380	0.799	
T-classification 1–2, *n* (%)	4 (25.0%)	3 (14.3%)	18 (50.0%)	64 (48.9%)	0.009		
Mean OS (months)	100	64.0 (25.5)	73.7 (9.7)	53.9 (6.0)	0.265	<0.001	0.951
Mean RFS (months)	100	71.7 (23.1)	51.2 (8.5)	70.7 (6.3)	0.537	0.164	
T-classification 3–4, *n* (%)	12 (75.0%)	18 (85.7%)	18 (50.0%)	67 (51.1%)	0.009		
Mean OS (months)	100	38.4 (5.4)	66.4 (10.2)	85.1 (4.1)	0.050	<0.001	0.951
Mean RFS (months)	66.7 (11.9)	45.2 (4.4)	67.3 (10.6)	79.7 (4.7)	0.388	0.164	
N-classification 0, *n* (%)	8 (50.0%)	8 (44.4%)	15 (44.1%)	53 (41.4%)	0.922		
Mean OS (months)	100	81.0 (16.5)	80.7 (9.9)	83.7 (4.9)	0.700	0.001	0.930
Mean RFS (months)	67.8 (14.1)	77.5 (19.5)	57.2 (11.4)	75.9 (5.7)	0.312	0.623	
N-classification >1, *n* (%)	8 (50.0%)	10 (55.6%)	19 (55.9%)	75 (58.6%)	0.922		
Mean OS (months)	100	25.8 (6.1)	61.5 (8.1)	62.8 (5.2)	0.260	0.001	0.930
Mean RFS (months)	74.0 (14.7)	35.8 (7.4)	57.6 (9.3)	76.7 (5.1)	0.569	0.623	
Grading (G) 1, *n* (%)	2 (16.7%)	1 (5.0%)	2 (6.3%)	7 (6.0%)	0.422		
Mean OS (months)	100	6.0 (0.0)	100	100	0.019	0.139	
Mean RFS (months)	100	6.0 (0.0)	74.0 (0.0)	84.3 (14.3)	0.146	0.662	
Grading (G) 2, *n* (%)	8 (66.7%)	15 (75.0%)	19 (59.4%)	82 (70.7%)	0.397		
Mean OS (months)	100	66.4 (13.0)	75.7 (9.1)	65.8 (4.8)	0.313	0.413	
Mean RFS (months)	67.0 (17.1)	69.3 (14.3)	67.1 (9.8)	75.1 (4.9)	0.578	0.762	
Grading (G) 3, *n* (%)	2 (16.7%)	4 (20.0%)	11 (34.4%)	27 (23.3%)	0.352		
Mean OS (months)	100	100	42.0 (5.8)	68.9 (8.9)	0.796	0.969	
Mean RFS (months)	100	37.0 (9.5)	35.0 (6.5)	72.7 (9.0)	0.879	0.532	
AJCC I/II, *n* (%)	10 (62.5%)	11 (50.0%)	10 (30.3%)	42 (32.8%)	0.053		
Mean OS (months)	100	89.1 (10.1)	91.8 (7.8)	91.1 (4.2)	0.829	<0.001	0.007, 5.563 (1.590–19.462)
Mean RFS (months)	71.4 (12.3)	87.1 (11.9)	64.5 (13.8)	78.5 (6.2)	0.397	0.396	
AJCC III/IV, N (%)	6 (37.5%)	11 (50.0%)	23 (69.7%)	86 (67.2%)	0.053		
Mean OS (months)	100	29.6 (6.1)	63.0 (8.8)	60.6 (5.0)	0.421	<0.001	0.007, 5.563 (1.590–19.462)
Mean RFS (months)	74.0 (14.7)	37.7 (6.4)	50.4 (7.7)	74.3 (5.0)	0.347	0.396	

## Data Availability

The data presented in this study are available on request from the corresponding author.
